# A case of sternal osteomyelitis caused by defective wound healing after surgery of gingival cancer

**DOI:** 10.1016/j.idcr.2023.e01682

**Published:** 2023-01-07

**Authors:** Norihisa Ichimura, Noriyuki Yamamoto, Yusuke Urata, Shohei Ikutomi, Hideharu Hibi

**Affiliations:** a Department of Oral and Maxillofacial Surgery, Nagoya University Hospital, Nagoya, Japan; b Department of Oral and Maxillofacial Surgery, Kariya Toyota General Hospital, Kariya, Japan; c Department of Oral and Maxillofacial Surgery, Nagoya University Graduate School of Medicine, Nagoya, Japan

**Keywords:** Sternal osteomyelitis, Defective wound healing, Gingival cancer

## Case presentation

**Fig. 1:**
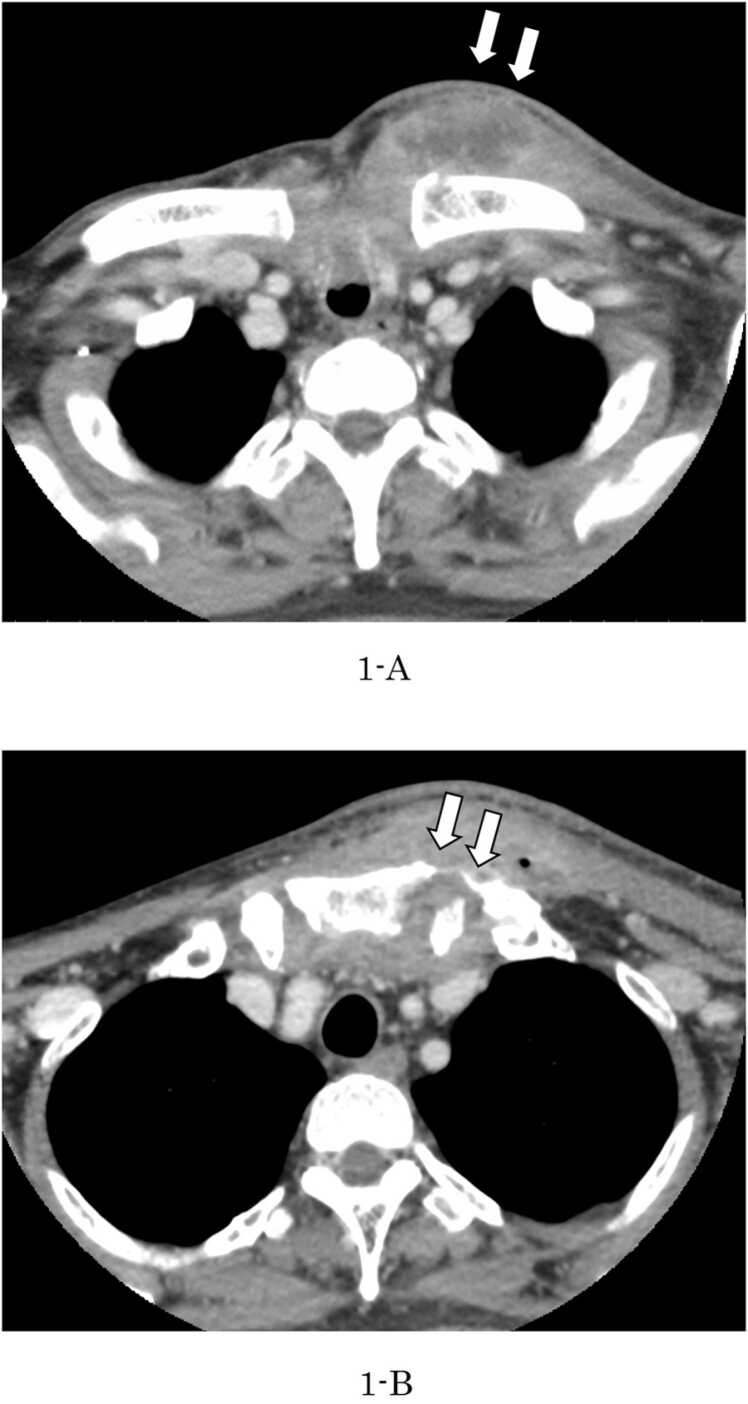
Computed tomography showed abscess formation around left clavicle (A), inside the sternum and an osteolytic region was observed on the left side of the sternal stalk (B), as indicated by the arrow.

A 50-year-old man with type 2 diabetes mellitus presented with marked swelling of the precordium. Two months earlier, he underwent tracheostomy, left neck dissection, segmental mandibulectomy, and reconstruction of the free peroneal composite flap with a vascular handle under general anesthesia at our department for left mandibular gingival cancer (cT4aN2bM0). During the physical examination, the patient was febrile (temperature of 100.8 ℉), his pulse was 107 beats/min, respirations 16 breaths/min, and oxygen saturation 97 % in room air. Laboratory testing revealed a white blood cell count of 11.1 × 10^3^/µL, C-reaction protein level of 11.74 mg/dL, and neutrophil count of 85.1 %. Extraoral examination revealed redness and swelling extending from above the left clavicle to the precordium. Computed tomography showed abscess formation around left clavicle, inside the sternum and an osteolytic region was observed on the left side of the sternal stalk (Fig. 1A, B). The patient was diagnosed as having sternal osteomyelitis and cervical cellulitis. On the same day, incision and pus drainage were performed under local anesthesia. Cefmetazole (4 g/day) was administered for three days. Subsequently, the patient was administered sulbactam/ampicillin (12 g/day) for 2 weeks according to the blood culture test, followed by potassium clavulanate and amoxicillin hydrate for 2 weeks. Twelve days after drainage, rotten bone removal of the sternum and clavicle, reconstruction with a pectoralis major flap, and skin grafting were performed under general anesthesia. Since then, the patient has continued to be followed up, and no recurrence of inflammation has been observed.

Sternal osteomyelitis is a relatively rare disease, and most are post-thoracotomy complications, so-called secondary sternal osteomyelitis [1], [2]. In this case, an abscess cavity was formed from the left side of the neck to the clavicle, continuing to the sternum, suggesting that the postoperative infection of the neck region had spread to the sternum. Hence, this case was considered secondary sternal osteomyelitis. During treatment, it is crucial to administer sensitive antibiotics. In cases with abscess formation, draining the pus early, and performing bone scraping together can lead to a good outcome [3]. The soft tissue in front of the sternum is thin, and infection tends to be intractable. Therefore, filling the dead space after lesion curettage requires reconstruction using soft tissue with abundant blood flow [4]. In this case, the patient underwent reconstruction using a pectoralis major flap and skin grafting. After more than two years of reconstruction, the patient was free from recurrence of sternal osteomyelitis.

## CRediT authorship contribution statement

**Norihisa Ichimura:** Conceptualization, Writing – original draft. **Noriyuki Yamamoto:** Supervision. **Yusuke Urata:** Writing – review & editing. **Shohei Ikutomi:** Writing – review & editing. **Hideharu Hibi:** Supervision.

## Sources of funding

This research did not receive any specific grant from funding agencies in the public, commercial, or not-for-profit sectors.

## Ethical approval

This case report was approved by the ethics committee of the Nagoya University Hospital, Nagoya, Japan.

## Consent

Written informed consent was obtained from the patient for publication of this case report and accompanying images.

## Conflict of Interest

The authors have no financial interests to disclose or conflicts of interest.
